# A research note: effect of pH on meat iridescence in precooked cured pork

**DOI:** 10.1186/s13104-022-05956-x

**Published:** 2022-02-22

**Authors:** Chiara Ruedt, Monika Gibis, Jochen Weiss

**Affiliations:** Department of Food Material Science, Institute of Food Science and Biotechnology, University of Hohenheim, Garbenstraße 21/25, 70599 Stuttgart, Germany

**Keywords:** Iridescence, Meat color, pH, Structural color

## Abstract

**Objective:**

The objective of this study was to investigate the effect of pH change of cooked cured pork *M. longissimus thoracis et lumborum* on iridescence intensity and extent (= percentage of iridescent area) since interaction with light may be related to pH-induced alterations in microstructure. Muscles were injected with brines of different pH values, cooked, sliced perpendicular to muscle fiber direction, and visually evaluated by a panel of 20 experienced panelists.

**Results:**

Muscles with lowest pH (5.38) showed the lowest iridescence score of 4.63 (p < 0.05). Iridescence was greatest in muscles with normal (5.78) and high pH (6.03, respectively 6.59), but did not differ significantly (p > 0.05). Iridescence was positively correlated (p < 0.01) with pH and water content, and negatively correlated (p < 0.01) with cooking loss. Hence, hydration state and light scattering from microstructure may be important factors that determine the degree of iridescence in cooked meat products.

## Introduction

The shimmering, rainbow-like colors from iridescence are a well-known but still not fully understood meat color phenomenon. Iridescence can be a problem for the meat industry due to consumers concerns about the quality and safety of iridescent green-colored meat causing a rejection of those products [1, 2]. Solution approaches to reduce or eliminate meat iridescence have been sought for many years but have been largely ineffective so far due to a lack of knowledge about the underlying mechanisms and structures. From a scientific point of view, iridescence is a fascinating physical phenomena that arises from coherent scattering of white incident light by transparent or semitransparent surface and subsurface structures [3]. In meat, iridescence is hypothesized to be caused by multilayer interference from the successive refractive index boundaries between A- and I-bands [4, 5]. Since the microstructure of the muscle strongly contributes to meat color by incoherent light scattering [6], meat iridescence was proposed to be a special case of light scattering in the long axis of myofibers [7]. Meat color is closely associated to water-holding capacity and pH [8–10]. Muscles with high pH have higher water-holding capacity, larger muscle fiber diameters, and longer distance between myofilaments allowing for more light to be transmitted into its interior causing meat to appear dark [11, 12]. Low pH, however, induces transverse shrinkage of the muscle fibers and myofilament lattice that causes both lower water-holding capacity and more incoherent scattering [11, 13]. Swatland [14] attributed iridescence to the hydration state of meat and interfilament spacing. Since the hydration state of meat and microstructural attributes causing scattering are interrelated with meat pH, the objective of this study was to investigate the effects of pH change on iridescence in cooked cured pork products.

## Main text

### Materials and methods

Fresh pork loins (*M. thoracis et lumborum*, n = 5) were purchased from a local wholesaler (Mega eG, Stuttgart, Germany). Each loin was divided into four equally sized pieces and randomly assigned to five different pH treatments: control, 0.2 M NaOH, 0.6 M NaOH, 1% lactic acid and 4% lactic acid. Injection brines were prepared by dissolving 15% (w/w) nitrite curing salt (0.4–0.5 g kg^−1^ NaNO_2_, Zentrag eG, Frankfurt, Germany) in water (= control) and adding lactic acid or sodium hydroxide to obtain a brine with 4% lactic acid, respectively 0.6 M NaOH. These higher concentrated brines were then diluted with the control brine to obtain the 1% lactic acid, respectively 0.2 M NaOH brines. pH values were measured with a puncture type pH probe (WTW SenTix Sp, Xylem Analytics Germany Sales GmbH & Co. KG, Weilheim, Germany) connected to a pH-meter (WTW pH 537, Xylem Analytics). The pH values of the brines were as follows: 7.37 (control), 12.07 (0.2 M NaOH), 12.13 (0.6 M NaOH), 2.23 (1% lactic acid), 1.57 (4% lactic acid). Muscles were injected (automatic pickle injector type PI 17, Günther Maschinenbau GmbH, Dieburg, Germany) with 15% (w/w) brine, individually vacuum-packaged (PA/PE 90 µm, Mega eG, Stuttgart, Germany) and tumbled (Vakona GmbH, Ditzingen, Germany) for 2 h at 2 °C. After a resting period of 12 h (2 °C), the muscles were cooked in a cooking chamber (Ness-Smoke GmbH & Co. KG, Remshalden, Deutschland) to a final core temperature of 70 °C (saturated steam, chamber temperature 74 °C). Cooking loss was calculated as the ratio of weight loss during cooking to the injected meat weight. Subsequently, muscles were cooled (12 h, 2 °C) and sliced (Type VS8A, Bizerba SE & Co. KG, Balingen, Germany) approximately 1 cm thick transversely to the longitudinal axis of the muscle fiber orientation. Each slice was individually vacuum-packaged to prevent drying and thereby reduction of surface iridescence and was evaluated visually by a trained sensory panel (n = 20, Meat Science Department of the University of Hohenheim) for iridescence intensity (ratio scale, 0 = no iridescence, 10 = very strong iridescence) and extent (ratio scale, 0 = no iridescence, 10 = 100% of surface affected). All panelists passed the Ishihara color test and were trained on the scale and the correct adjustment of sample rotation, observation and illumination angle to evaluate maximum iridescence. Iridescence score was calculated as the arithmetic mean of the intensity and extent and scores from the 20 panelists were averaged to give one score per slice. For proximate analysis, remaining muscles were finely comminuted (Blixer 2, Robot Coupe SA, France). Water, ash and sodium chloride content were determined according to the official collection of methods of analysis (§64 German Food and Feed Act, LFGB) [15]. Protein content was measured with rapid nitrogen analysis according to the Dumas method (§64 L 01.00-60) [15] with the Dumatherm N Pro (Gerhardt GmbH & Co. KG, Königswinter, Germany) calibrated with EDTA (ethylenediaminetetraacetic acid, ≥ 99%, p.a, ACS, Carl Roth GmbH + Co. KG, Karlsruhe, Germany) A nitrogen to protein conversion factor of 6.25 for meat was used. Each determination was performed in duplicate.

Statistical analysis was performed with SPSS (IBM SPSS Statistics, Version 25.0, IBM Corp. Released 2017, IBM Corp., Armonk, NY, USA) and OriginPro (OriginPro, Version 2018b, OriginLab Corporation, Northampton, MA, USA) software. Assumptions of normal distribution and homogeneity of variance were tested with Shapiro Wilk and Brown-Forsythe test. Data that met both assumptions were analyzed with one-way ANOVA (treatment as factor). Fisher’s LSD post hoc test was calculated to test for differences between the treatments means. Data that did not meet the assumption of homoscedasticity were analyzed with a Welch ANOVA and Games-Howell post hoc test. Pearson correlation coefficient (r) was calculated to measure the dependence between iridescence and physicochemical properties. A significance level of α = 0.05 was used.

### Results and discussion

**Fig. 1:**
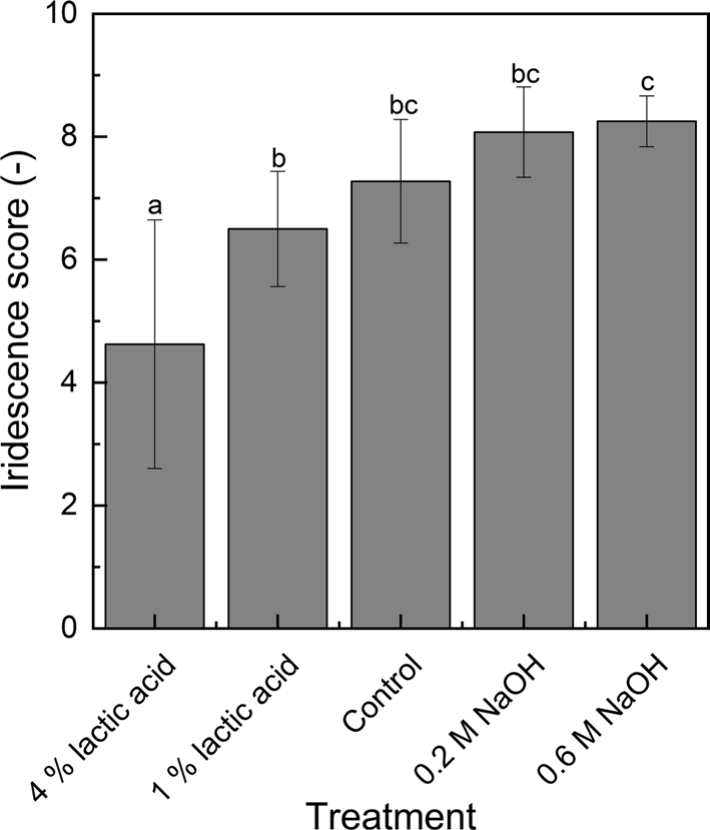
Average visual scores (arithmetic mean of extent and intensity) of iridescence (n = 20) in cooked cured pork *M. longissimus dorsi* (n = 4) as influenced by different pH brine treatments. Bars without a common superscript differ significantly (p < 0.05)

All samples showed iridescence. The lowest iridescence score was observed in the 4% lactic acid treatment with the lowest ultimate pH (Fig. 1). Strongest iridescence was observed in the control samples and samples injected with 0.2 M, respectively 0.6 M NaOH. No significant differences (p > 0.05) in iridescence scores were found between the 1% lactic acid treatment, control and 0.2 M NaOH treatment as well as between the control and the sodium hydroxide treatments. Both iridescence intensity and extent increased with higher ultimate pH values (Table 1). Lowest intensity and extent was found in the lactic acid treatments. Iridescence extent was only evaluated significantly lower in the 4% lactic acid treatment. Raw meat pH values were similar in the range of 5.36 ± 0.53 to 5.44 ± 0.11 and injection of the different brines lowered the pH values of the cooked muscles to 5.38 ± 0.03, or increased the pH to 6.59 ± 0.22. A moderate positive correlation (r = 0.645, p < 0.01) was found between iridescence score and pH of the cooked muscles. Interestingly, the pH values of the control and the 1% lactic acid treatment did not differ significantly which may explain the similar degree of iridescence. However, also the 0.2 M NaOH treatment with a significantly higher pH (6.03 ± 0.09) showed similar iridescence. Water content also showed a moderate positive (r = 0.601, p < 0.01) and cooking loss a strong negative correlation (r = -0.728, p < 0.01) with iridescence. Wang [2] reported a weak relationship of iridescence with pH and water in cooked beef muscles and Kukowski, Wulf [16] found a moderate negative correlation between iridescence and ultimate pH, and no correlation with cooking loss. However, in their studies pH values were in a narrow range and it seems likely that the differences were not sufficient to have a sustained effect on hydration state and thus on iridescence. Water-holding is determined by the net charge of the myofibrillar proteins that cause an electrostatic repulsion between the myofilaments [17]. The degree of swelling of the microstructure is a function of the pH. At a low pH close to the average isoelectric point of the myofibrillar proteins at 5.0 [18] a shrinkage of the structures (fibers, myofilaments, myofilament lattice) occurs and light scattering increases [11, 12]. According to Hughes, Clarke [19] the formation of extracellular space from transverse shrinkage of muscle fibers is believed to be the major cause for increased light scattering. It may be the case therefore that iridescence decreased at a low pH due to transversal shrinkage of the structural attributes causing larger extracellular spaces and stronger incoherent scattering of the incoming light that thus suppressed light interference. These results provide further support for the hypothesis of Swatland [20] that a high pH allows iridescence to appear because of less scattering from myofibrillar refraction. However, it must be noted that a significant effect on iridescence was only observed for relatively high pH differences and even the samples with low pH showed moderate to strong iridescence. Additionally, differences in iridescence (both extent and intensity) were observed between the replicates within the treatments indicating that iridescence is not just influenced by the production process or treatment but also by the raw material characteristics that were not investigated in this study. It is likely that fresh muscle characteristics such as the ultimate pH, water-binding capacity and in particular the myofibers orientation strongly impact the appearance and strength of iridescence. As previously discussed, lower ultimate muscle pH would cause a lower water-binding capacity and thus a stronger shrinkage of muscle fibers and stronger incoherent light scattering that overlays the meat iridescence. In general, therefore, it seems that the occurrence of iridescence and muscle-to-muscle variations result from a complex interaction between physicochemical properties and microstructural attributes and that iridescence is an inherent characteristic of meat related to the highly ordered and hierarchical structure of cross-striated muscles.

**Table 1:** Results from sensory and physicochemical analysis for cooked cured pork *M. longissimus dorsi* (n = 4) injected with different pH brines

	Treatment					Correlation coefficient r
	4% lactic acid	1% lactic acid	Control	0.2 M NaOH	0.6 M NaOH	
Intensity (–)	3.5 ± 2.0^b^	5.4 ± 1.4^bcd^	6.0 ± 1.2^ad^	7.3 ± 1.3^ac^	7.8 ± 0.7^a^	0.670*
Extent (–)	5.8 ± 2.1^a^	7.7 ± 1.1^b^	8.5 ± 0.9^b^	8.9 ± 0.3^b^	8.8 ± 0.4^b^	0.567*
pH raw (–)	5.40 ± 0.61^a^	5.36 ± 0.53^a^	5.39 ± 0.07^a^	5.44 ± 0.11^a^	5.43 ± 0.04^a^	0.115
pH cooked (–)	5.38 ± 0.03^a^	5.64 ± 0.04^b^	5.78 ± 0.06^b^	6.03 ± 0.09^c^	6.59 ± 0.22^d^	0.645*
Cooking loss (%)	32.5 ± 2.8^c^	24.6 ± 1.9^a^	19.9 ± 3.9^a^	10.5 ± 2.0^b^	6.7 ± 0.3^b^	− 0.728*
Water (%)	64.43 ± 2.54^c^	67.85 ± 1.03^d^	70.23 ± 1.07^a^	71.65 ± 0.93^ab^	72.65 ± 1.25^b^	0.601*
Protein (%)	30.15 ± 2.58^b^	25.87 ± 2.10^c^	23.39 ± 1.20^ cd^	21.71 ± 2.69^d^	22.81 ± 0.62^d^	− 0.647*
Ash (%)	2.20 ± 0.10^c^	2.79 ± 0.18^a^	2.85 ± 0.13^ab^	3.07 ± 0.18^bd^	3.27 ± 0.15^d^	0.750*
NaCl (%)	1.39 ± 0.14^b^	1.94 ± 0.22^a^	1.94 ± 0.14^a^	2.08 ± 0.18^a^	2.14 ± 0.17^a^	0.696*

Pearson correlation coefficient r between intensity and extent with pH of cooked samples, respectively between iridescence score and physicochemical measurements. Data points within a row with different superscripts differ significantly (p < 0.05)

^*^Significant correlation α = 0.01 (two-tailed)

## Limitations

The purpose of this research was to investigate the influence of pH on meat iridescence in cured cooked pork meat. Iridescence was positively correlated with pH and water content, and negatively correlated with cooking loss. These findings support the hypothesis that iridescence is a special case of light scattering along the myofibers and that incoherent scattering from structural attributes suppresses iridescence. A major limitation of this study is the lack of information on the raw fresh muscle characteristics since it can be assumed that these parameters have a crucial impact on meat iridescence. Thus, it must be kept in mind that differences in iridescence might also arise from differences in the fresh muscle characteristics. In terms of possible solutions, there seems to be very limited approaches to reduce the potential problem of iridescence since lower hydration results in lower yields and reduced quality of processed meats.

## Data Availability

The datasets used and analysed during the current study are available from the corresponding author on reasonable request.
